# SEZ6L2 knockdown impairs tumour growth by promoting caspase‐dependent apoptosis in colorectal cancer

**DOI:** 10.1111/jcmm.15082

**Published:** 2020-02-27

**Authors:** Ning An, Yaqin Zhao, Haitao Lan, Ming Zhang, Yuan Yin, Cheng Yi

**Affiliations:** ^1^ Department of Abdominal Cancer West China Hospital West China Clinical Medical School Sichuan University Chengdu China; ^2^ Cancer Center Academy of Medical Sciences and Sichuan Provincial People's Hospital Affiliated Hospital of University of Electronic Science and Technology of China Chengdu China; ^3^ Department of Gastrointestinal Surgery West China Hospital and State Key Laboratory of Biotherapy Sichuan University Chengdu China

**Keywords:** apoptosis, caspase, colorectal cancer, mitochondria, prognosis, SEZ6L2

## Abstract

Seizure‐related 6 homolog (mouse)‐like 2 (SEZ6L2) was shown to be involved in transcription of a type 1 transmembrane protein for regulating cell fate. Until now, the expression and function of SEZ6L2 in various cancers, including colorectal cancer (CRC), were unclear. In the present study, we determined the expression of SEZ6L2 in a tissue microarray from patients with CRC and then, analysed the correlation between SEZ6L2 expression and the prognosis of the patients. Furthermore, the potential function of SEZ6L2 in CRC was determined using cell counting kit, colony formation assay and xenograft model in vitro and in vivo. Flow cytometry, Western blotting, immunohistochemical staining and a blocking experiment were employed to investigate the underlying mechanism of SEZ6L2 regulating CRC growth. Our results indicated that SEZ6L2 was significantly up‐regulated in tumour tissues of patients with CRC compared with adjacent normal tissues. Up‐regulation of SEZ6L2 was correlated with a poor prognosis in patients with CRC. In vitro experiments suggested that the knockdown of SEZ6L2 inhibits CRC cell growth and colony formation, but it has no significant impact on the invasion. The antitumour effects of shSEZ6L2 were also confirmed by a xenograft model. Investigations of the mechanisms indicated that the knockdown of SEZ6L2 impairs the growth of the CRC cells by inducing caspase‐dependent apoptosis, which was mediated by mitochondria‐related proteins. Furthermore, SEZ6L2 expression was inversely correlated with the expression of cytochrome C in malignant tissues in patients with CRC. Collectively, the present study indicates that SEZ6L2 is a potential prognosis biomarker and therapy target for CRC.

## 1 INTRODUCTION

Colorectal cancer (CRC) has been a common malignancy and leading cause of cancer mortality in the developed and developing countries, in recent years.^1^ Epidemiological data in recent years suggest that the 5‐year prevalence of CRC has reached 74.6 and 58.3 per 100 000 men and women, respectively.^2^ According to the data, approximately 41% of all CRCs occur in the proximal colon, while 22% occur in the distal colon and 28% in the rectum.^2^ With the advancements in understanding the pathogenesis, CRC is typically seen as a series of mutations and epigenetic changes that accumulate slowly, which may lead to a loss of function in tumour‐suppressor genes and an increase in oncogenes.^3^, ^4^, ^5^ Thus, understanding the function and mechanism of hub genes during CRC development could provide potential prognosis biomarkers and therapy targets for CRC.^6^, ^7^

Seizure‐related 6 homolog (mouse)‐like 2 (SEZ6L2), a type 1 transmembrane protein predominantly expressed in the brain, belongs to the seizure‐related gene 6 (SEZ6) family, which contains SEZ6, SEZ6L and SEZ6L2. It has been reported that mice deficient in SEZ6L2 suffer from motor ataxia, impaired cognition and abnormal neuronal innervation (a disturbance of the cerebellar synaptic maturation in mutant mice lacking BSRPs, a novel brain‐specific receptor‐like protein family).^8^ Cleavage of SEZ6L2, which is mediated by cathepsin D, induces neurite outgrowth.^9^ In one patient suffering from cerebellar ataxia and retinopathy, anti‐SEZ6L2 antibodies were detected,^10^ which were also associated with progressive cerebellar ataxia.^11^ In non‐small‐cell lung cancers (NSCLC), patients whose tumours revealed a higher level of SEZ6L2 expression suffered shorter tumour‐specific survival compared to those with no SEZ6L2 expression.^12^ However, the expression and function of SEZ6L2 in CRC was still unclear.

In the present study, we aimed to investigate the expression and function of SEZ6L2 in CRC. A tissue microarray (TMA) containing 160 tissues from patients with CRC was performed for SEZ6L2 determination using immunohistochemical (IHC) staining, followed by an analysis of the correlation between SEZ6L2 expression and prognosis of the patients. Furthermore, the potential function of SEZ6L2 in CRC was determined by a cell counting kit, colon formation assay, and xenograft model in vitro and in vivo. Flow cytometry, Western blotting, IHC staining, and a blocking experiment were employed to investigate the underlying mechanism of SEZ6L2 regulating CRC growth. Collectively, these investigations would provide solid evidence for understanding the potential function and predicting role of SEZ6L2 in CRC.

## 2 MATERIALS AND METHODS

### 2.1 Cell culture and treatment

The CRC cell lines and human intestinal epithelial cell (HIEC) were obtained from the American Type Culture Collection (ATCC). The cells were cultured in low‐glucose Dulbecco's modified Eagle's medium (Thermo Fisher Scientific, Inc) with 10% foetal bovine serum (Thermo Fisher Scientific, Inc), 1% antibiotics (100 U/mL penicillin; 100 U/mL streptomycin) and 5 µmol/L 2‐Mercaptoethanol (Sigma‐Aldrich Co.). The cells were infected with lentivirus‐shSEZ6L2 (GenePharma), and the stable infected cells were selected by puromycin (2 mg/mL, Selleck). The z‐VAD‐FMK, dissolved in DMSO (Sigma‐Aldrich Co.) were added to treat the cells. All cells cultured in the atmosphere containing 5% CO_2_ at 37°C for 2 weeks.

### 2.2 Western blotting

The cells were lysed using RIPA buffer (20 mmol/L Tris‐HCl, pH 7.5, 200 mmol/L NaCl, 1% Triton X‐100, 1 mmol/L dithiothreitol) containing protease inhibitor cocktail (Merck Millipore). Protein concentrations were measured with a BCA assay kit (Thermo Fisher Scientific), and proteins were then separated by SDS‐PAGE and transferred to polyvinylidene difluoride membranes (Merck Millipore). The membranes were then sequentially incubated with the appropriate primary antibodies (SEZ6L2, caspase 3, caspase 8, caspase 9, Bim, Bcl‐2, Bax, BID, Cyto C (Cell Signaling Technology) and horseradish peroxidase‐conjugated secondary antibody. The ECL kit (Merck Millipore) was used to detect the specific bands with iBright 2000 (Thermo Fisher). GAPDH, and COX IV was used as a loading control.

### 2.3 Cell viability assay and colony formation assay

The cell viability assay and colony formation assay were performed as previous study indicated.^13^ Briefly speaking, the cells were seeded at 1 × 10^3^ cells per well in 96‐well plates. At 0, 24, 48 and 72 hours after cells plating, 10 μL Cell Counting Kit‐8 (CCK‐8; Dojindo) was added into each well, and the absorbance was measured at 450 nm at different time points with microplate reader (Thermo Fisher Scientific). For colony formation assay, the cells were plated into six‐well plates (1000 cells per well) and cultured for approximately 14 days. And then, after crystal violet staining, the colony formed in each well was counted and analysed.

### 2.4 Invasion assay

Cell invasion was determined using a 8 μm Transwell cell culture chamber (Merck Millipore). After starvation in serum‐free DMEM for 24 hours, 1 × 10^4^ cells were added to the upper chamber with serum‐free DMEM and incubated for 24 hours in the presence of 20% FBS in the lower chamber. The cells at the reverse side of a filter were fixed with 4% poly‐paraformaldehyde and stained with crystal violet. The cells were photographed with BX51 (Olympus), and the number of cells in each frame was counted and analysed.

### 2.5 Flow cytometry

The apoptosis of CRC cells was detected by Annexin V‐FITC/PI kit (Keygentec), following the instructions of manufacturers. Flow cytometry analysis was performed with BD LSRII Flow Cytometer (Becton Dickson), and data were analysed by BD FACSDiva software.

### 2.6 Animal study

For the xenograft model, stably infected HCT116 and HT29 cells were injected subcutaneously into the flank of 6‐8 weeks old female Balb/c nude mice (Hfkbio). The width and length of xenografts were measured every five days. The tumour volume was calculated as volume = length × width^2^ × 0.52. All mice were kept in a specific pathogen free (SPF) room, with free access to food and water.

### 2.7 Terminal‐deoxynucleoitidyl Transferase Mediated Nick End Labelling (TUNEL)

DeadEndTM Fluorometric TUNEL System (Promega) was used to detect the apoptotic cells following the manufacturer's protocol. 4,6‐diamidino‐2‐phenylindole (DAPI, Beyotime) was employed to detect the cell nuclei. The cell nuclei with dark green fluorescent staining were defined as apoptotic cells. The slides were photographed with BX51 (Olympus), and the number of cells in each frame was counted and analysed.

### 2.8 Immunohistochemical staining

Tissue Micro Array (TMA) containing 160 CRC tissues was purchased from Outdo Bio. For IHC staining of the xenografts, tumour tissues were fixed, embedded and sectioned (4 μm thick). Immunohistochemistry (IHC) staining for TMA and xenograft tissues was performed in accordance with previous procedures.^14^ The following antibodies were used as follows: primary antibody against SEZ6L2 (1:100) or cytochrome C (1:100) (Cell Signaling Technology) was used. The staining was scored as follows: 0, the per cent of positive cells < 5%; 1, 5% < the per cent of positive cells < 25%; 1, 25% < the per cent of positive cells < 45%; 1, 45% < the per cent of positive cells. All of the staining was blind evaluated by two specialized staff in pathology.

### 2.9 The cancer genome atlas (TCGA)‐based analysis

The SEZ6L2 mRNA expression data in 470 CRC tissues and 41 normal colon tissues were downloaded from TCGA database, accompanied with the data of overall survival time of patients. The patients were also classified into two groups, high SEZ6L2 expression and low SEZ6L2 expression, based on the average expression of all patients. The Kaplan‐Meier method was employed to estimate the survival curves in two group patients in 12 years.

### 2.10 Statistical analysis

Statistical analysis was performed using the SPSS Statistics software package (IBM, standard version 22.0). All of the data were performed as mean ± standard deviation. The differences were analysed by Student's *t* test or one‐way analysis. The Kaplan‐Meier method was employed to estimate the survival curves, followed with the long‐rank test for comparing the difference. The correlation was analysed by Pearson's analysis. *P* value < .05 was considered to indicate statistical significance.

## 3 RESULTS

### 3.1 Up‐regulation of SEZ6L2 correlates with poor prognosis for patients with CRC

**Figure 1:**
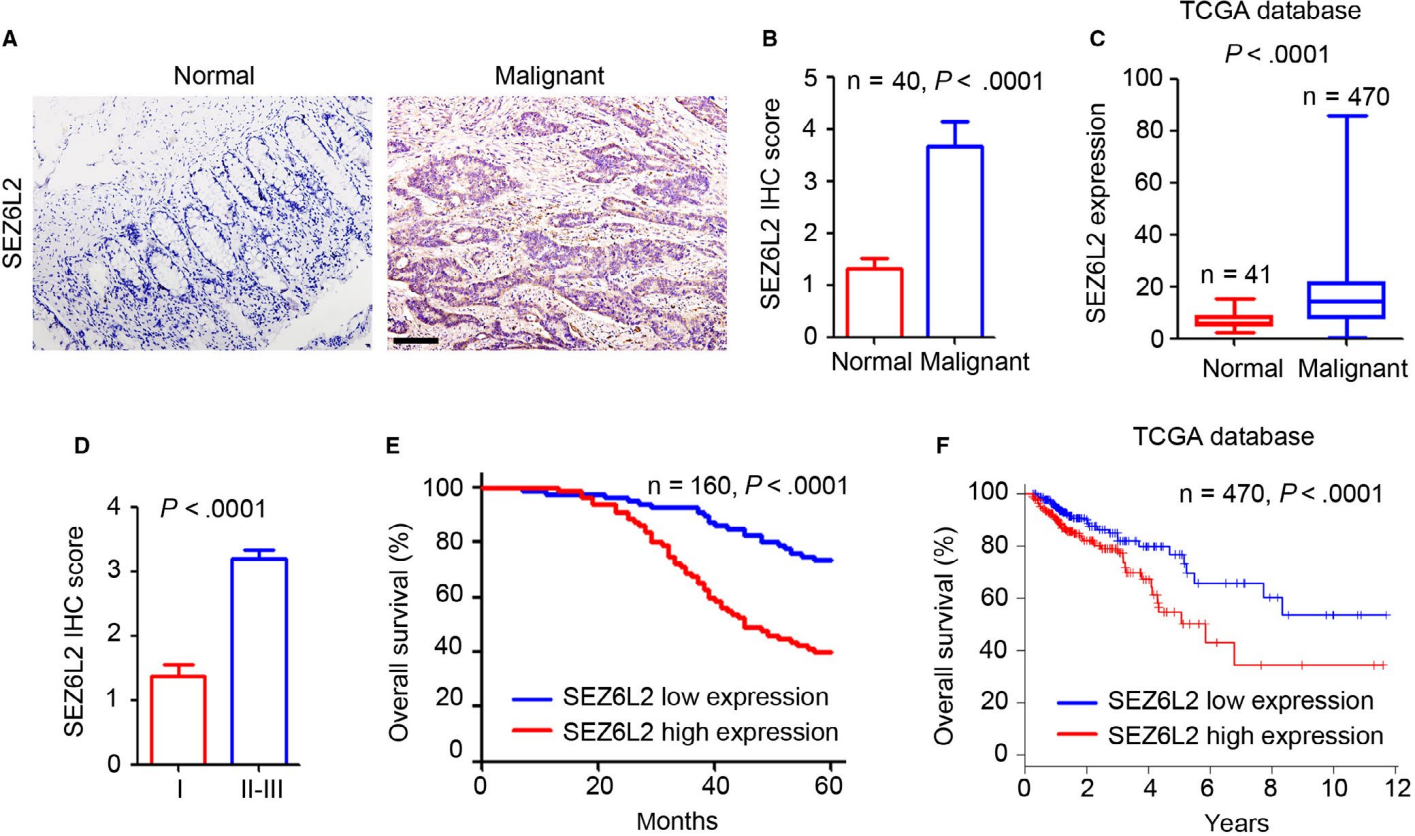
Up‐regulation of SEZ6L2 correlates with poor prognosis of CRC patients. A, IHC staining of SEZ6L2 expression in two tumour microarray containing normal and malignant tissues of CRC patients. Scale bar = 100 μm. B, Scoring of SEZ6L2 expression based on the IHC staining. Analysis of the SEZ6L2 expression in 40 pairs of normal and malignant tissues. C, Analysis of the SEZ6L2 mRNA expression in 41 normal tissues and 470 malignant tissues based on the TCGA database. D, Analysis of the SEZ6L2 expression in malignant tissues of phase I and phase II‐III CRC patients. E, Kaplan‐Meier curve showing overall survival of CRC patients, stratified by SEZ6L2 expression (high‐ and low‐scoring tumours) based on the IHC score. F, Kaplan‐Meier curve showing overall survival of CRC patients, stratified by SEZ6L2 mRNA expression (high‐ and low‐scoring tumours) based on the TCGA database

To investigate the expression of SEZ6L2 in CRC tissues, two tissue microarrays, one containing 160 CRC tissues and the other containing 40 normal adjacent tissues, were performed for SEZ6L2 detection using immunohistochemical (IHC) staining. As shown in Figure 1A, fewer SEZ6L2‐positive cells were observed in the normal tissues, whereas SEZ6L2 was highly expressed in the malignant tissues of patients with CRC. Further, IHC score analysis confirmed the significant up‐regulation of SEZ6L2 in malignant tissues compared with normal tissues (Figure 1B). Analysis of the TCGA database indicated that the expression of SEZ6L2 mRNA was also dramatically up‐regulated in malignant tissues (Figure 1C). Based on the IHC score, the results also showed higher expression of SEZ6L2 in the malignant tissues of patients in phase II‐III, compared with patients in phase I (Figure 1D). The patients were also classified into two groups, high SEZ6L2 expression and low SEZ6L2 expression, based on their IHC scores. Further analysis demonstrated that the patients with low SEZ6L2 expression had a higher percentage of 5‐year overall survival rates (Figure 1E). The TCGA database results also confirmed the positive correlation between SEZ6L2 expression and poor prognosis in patients with CRC (Figure 1F). Collectively, the results suggested that SEZ6L2 is up‐regulated in CRC tissues and correlates with poor prognosis for patients.

### 3.2 SEZ6L2 promotes CRC cell growth in vitro

**Figure 2:**
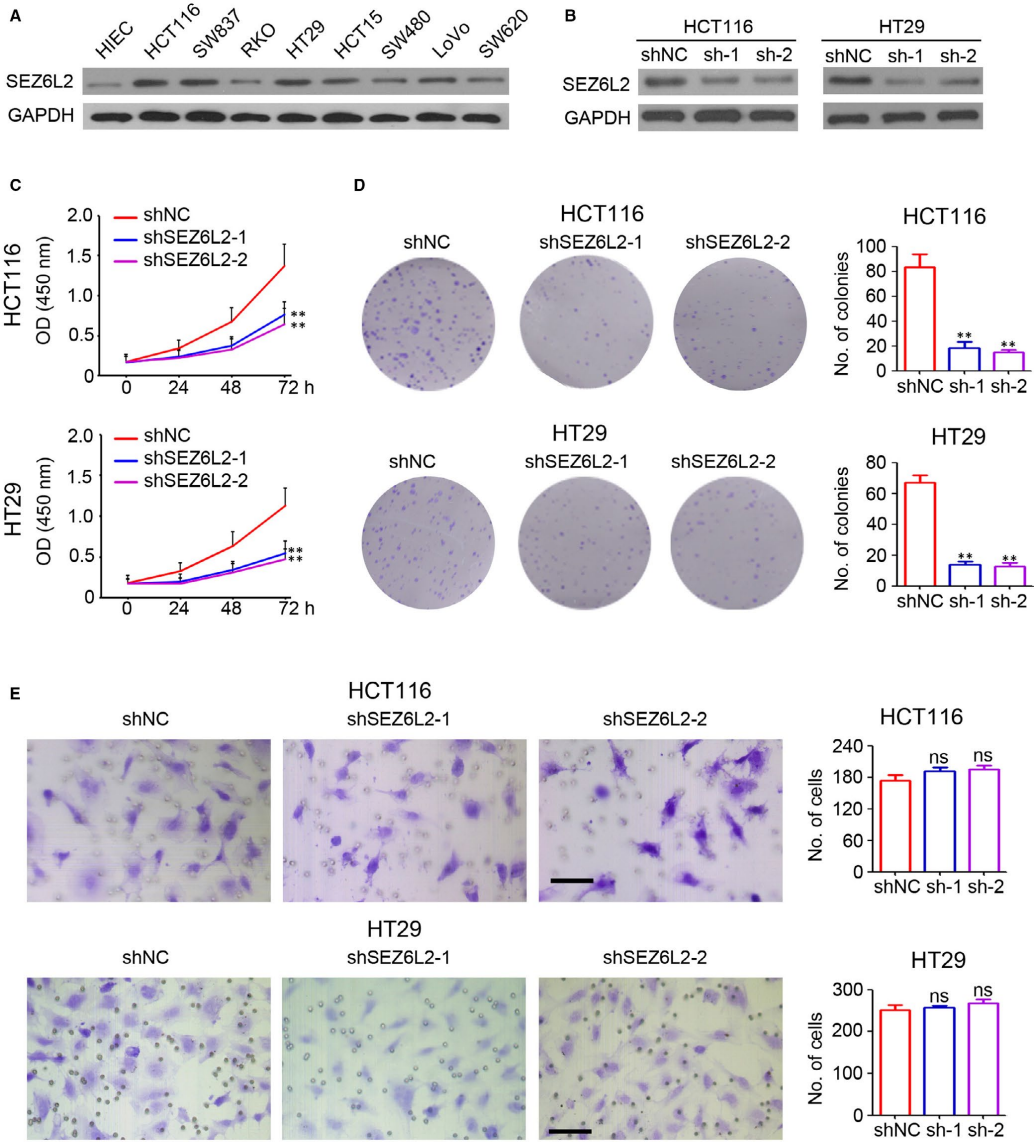
SEZ6L2 promotes CRC cell growth in vitro. A, Western blotting analysis of SEZ6L2 expression in human intestinal epithelial cells (HIEC) and CRC cells lines. GAPDH was used as a loading control. B, Western blotting analysis of SEZ6L2 expression in HCT116 and HT29 cells that were stably infected with lenti‐shSEZ6L2‐1 and lenti‐shSEZ6L2‐2. GAPDH was used as a loading control. C, Detection of cell viability at 0, 24, 48 and 72 h after cell plating by CCK‐8 assay. The absorbance of OD450 nm was recorded (n = 4, **P < .01). D, Colony formation analysis of HCT116 and HT29 cells that were stably infected with lenti‐shSEZ6L2‐1 and lenti‐shSEZ6L2‐2 (14 d after cell plating). The number of colonies in each well was counted and analysed (n = 4, **P < .01). E, Invasion analysis of HCT116 and HT29 cells that were stably infected with lenti‐shSEZ6L2‐1 and lenti‐shSEZ6L2‐2. The number of invaded cells in each well was counted and analysed (n = 4, ns, no significant difference)

Next, we aimed to determine the functional role of SEZ6L2 in CRC. The expression of SEZ6L2 in several CRC cell lines and human intestinal epithelial cells (HIEC) was detected by Western blotting. Our results indicated that SEZ6L2 was highly expressed in all detected CRC cells, including HCT116 and HT29 (Figure 2A). Thus, lentivirus‐based shRNA targeting SEZ6L2 was employed to infect HCT116 and HT29 cells and the stable infected cells were selected by adding puromycin. Western blotting results confirmed the efficient knockdown of SEZ6L2 in HCT116 and HT29 cells that were infected with lenti‐shSEZ6L2‐1 or lenti‐shSEZ6L2‐2 (Figure 2B). The results of the CCK‐8 assay suggested that the knockdown of SEZ6L2 significantly inhibited the growth of HCT116 and HT29 cells (Figure 2C). Furthermore, fewer colonies were formed in the HCT116 and HT29 cells that were infected with lenti‐shSEZ6L2‐1 or lenti‐shSEZ6L2‐2 (Figure 2D). The invasion assay indicated that the knockdown of SEZ6L2 has no significant effect on the invasion ability of CRC cells (Figure 2E). Collectively, the above results suggested that the knockdown of SEZ6L2 inhibits CRC cell growth in vitro.

### 3.3 Knockdown of SEZ6L2 promotes apoptosis by regulating mitochondria‐related protein expression in CRC

**Figure 3:**
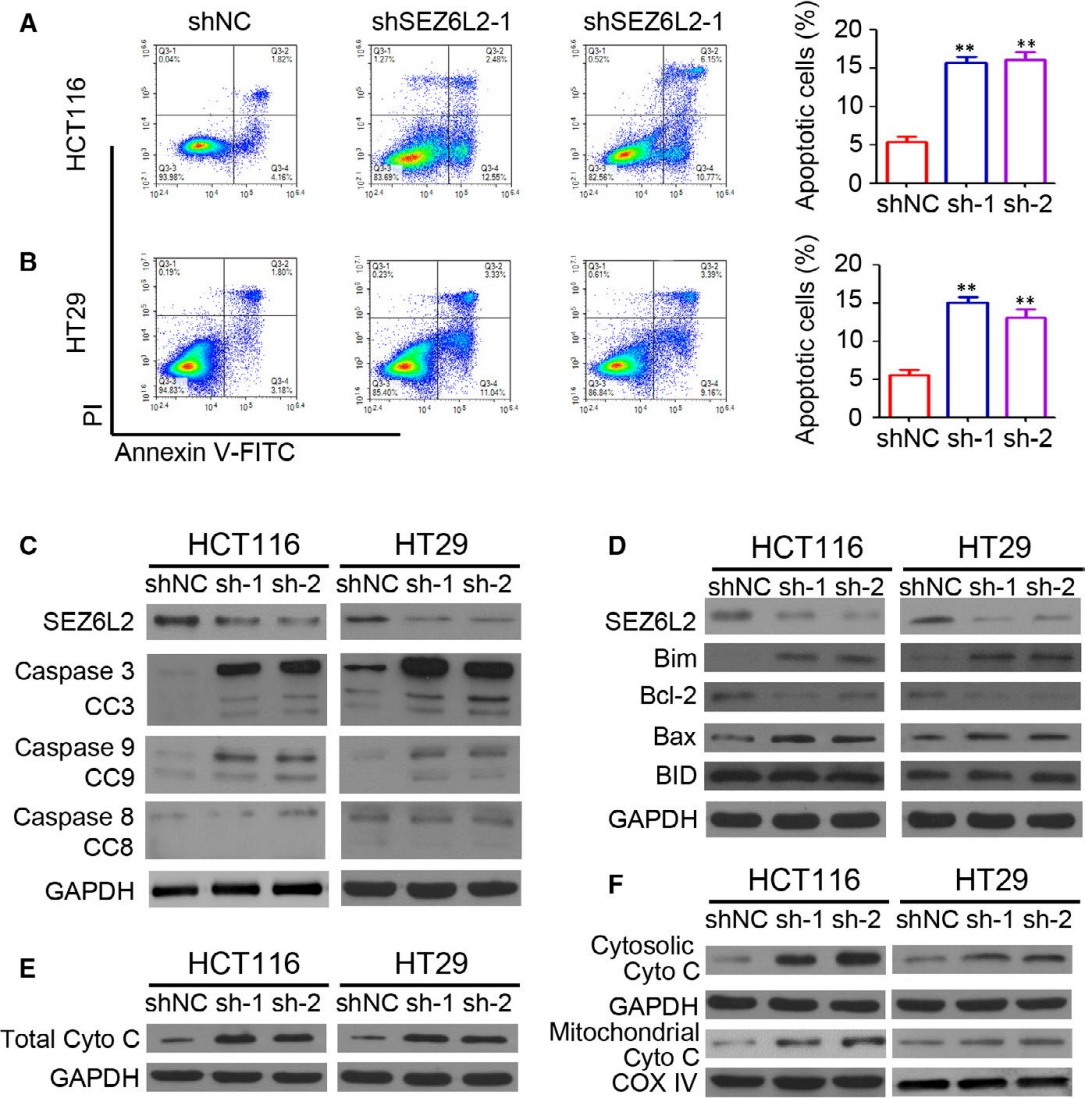
Knockdown of SEZ6L2 promotes apoptosis through regulating mitochondria‐related protein expression in CRC. A and B, Detection of apoptosis cells by flow cytometry in HCT116 (A) and HT29 (B) cells that were stably infected with lenti‐shSEZ6L2‐1 and lenti‐shSEZ6L2‐2. The percentage of apoptotic cells were analysed (n = 4, **P < .01). C, Western blotting analysis of caspase 3, cleaved caspase 3 (CC3), caspase 8, cleaved caspase 8 (CC8), caspase 9 and cleaved caspase 9 (CC9) expression in HCT116 and HT29 cells that were stably infected with lenti‐shSEZ6L2‐1 and lenti‐shSEZ6L2‐2. GAPDH was used as a loading control. D, Western blotting analysis of Bim, Bcl‐2, Bax and BID expression in HCT116 and HT29 cells that were stably infected with lenti‐shSEZ6L2‐1 and lenti‐shSEZ6L2‐2. GAPDH was used as a loading control. E, Western blotting analysis of total cytochrome C expression in HCT116 and HT29 cells that were stably infected with lenti‐shSEZ6L2‐1 and lenti‐shSEZ6L2‐2. F, Western blotting analysis of cytosolic and mitochondrial cytochrome C expression in HCT116 and HT29 cells that were stably infected with lenti‐shSEZ6L2‐1 and lenti‐shSEZ6L2‐2. GAPDH was used a cytosolic loading control. COX IV was used mitochondrial loading control

To investigate the potential underlying mechanism of SEZ6L2 regulating CRC growth, the HCT116 and HT29 cells that were infected with lenti‐shSEZ6L2‐1 or lenti‐shSEZ6L2‐2 were collected for apoptosis detection by flow cytometry. Our results indicated that the knockdown of SEZ6L2 significantly promotes apoptosis, both in HCT116 (Figure 3A) and HT29 (Figure 3B) cells. Furthermore, Western blotting was used to detect caspase protein expression. As shown in Figure 3C, greater total and cleaved caspase 3 and 9 protein expression was observed in HCT116 and HT29 cells that were infected with lenti‐shSEZ6L2‐1 or lenti‐shSEZ6L2‐2. The knockdown of SEZ6L2 had no significant effect on the expression of total and cleaved caspase 8 in CRC cells (Figure 3C). Furthermore, the knockdown of SEZ6L2 promotes the expression of the pro‐apoptosis proteins Bim, and Bax in HCT116 and HT29 cells (Figure 3D), whereas it inhibits the expression of the anti‐apoptosis protein Bcl‐2 (Figure 3D). Notably, SEZ6L2 has no observed effect on BID expression in CRC cells (Figure 3D). Further results also suggested the up‐regulation of total cytochrome C in SEZ6L2 knockdown cells (Figure 3E), accompanied with increasing of cytosolic cytochrome C and mitochondrial cytochrome C (Figure 3F). The above results suggest that the knockdown of SEZ6L2 promotes apoptosis by regulating mitochondria‐related protein expression in CRC.

### 3.4 Knockdown of SEZ6L2 promotes caspase‐dependent apoptosis in CRC

**Figure 4:**
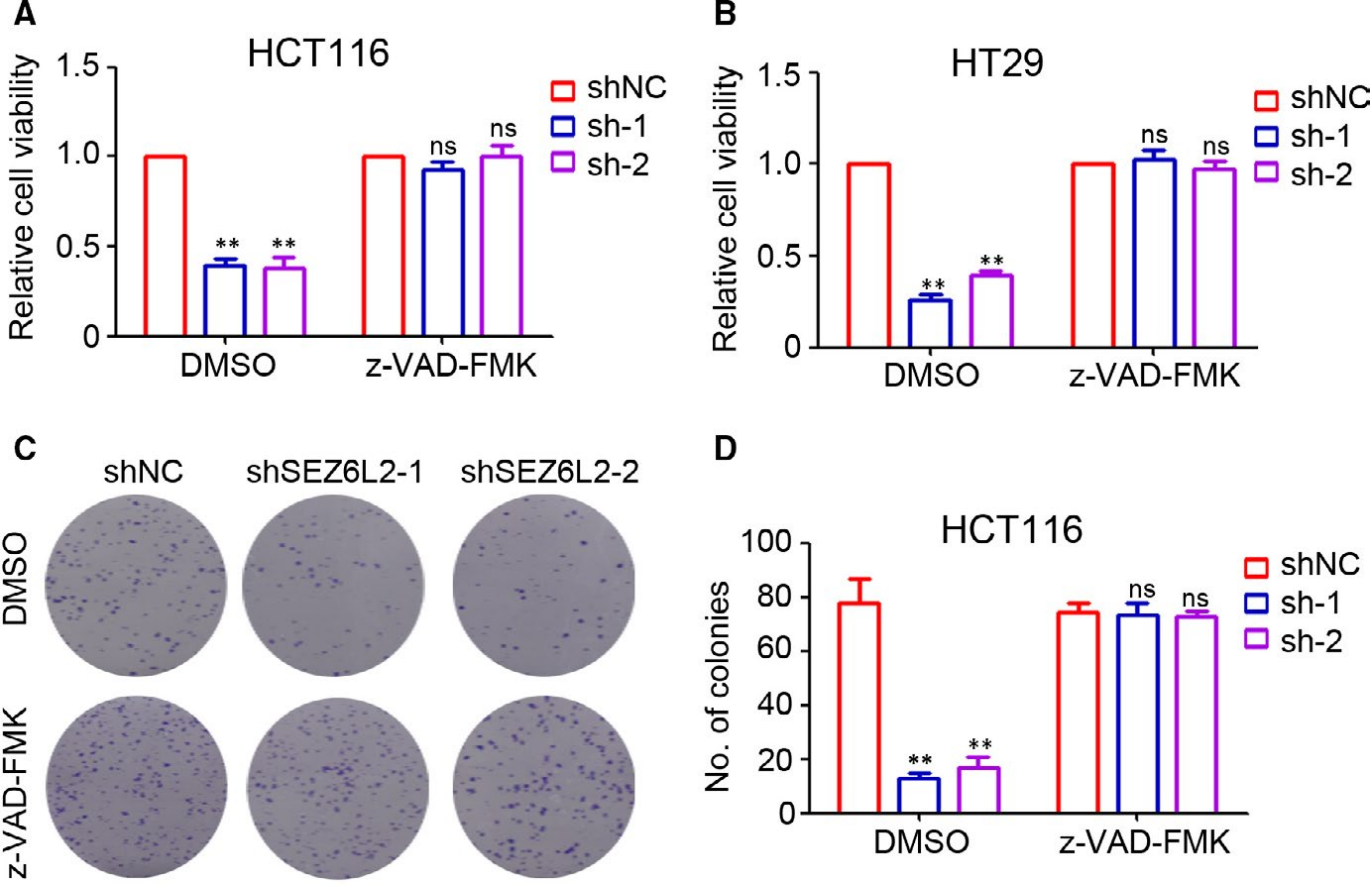
Knockdown of SEZ6L2 promotes caspase‐dependent apoptosis in CRC. A and B, Detection of cell viability at 72 h after cell plating by CCK‐8 assay. The relative cell viability was analysed. (n = 4, **P < .01, compared with shNC‐DMSO group; ns, no significant difference, compared with shNC‐z‐VAD‐FMK group). C and D, Colony formation analysis of HCT116 cells that were treated with DMSO and z‐VAD‐FMK (14 d after cells plating). The number of colonies in each well was counted and analysed (n = 4, **P < .01; compared with shNC‐DMSO group; ns, no significant difference, compared with shNC‐z‐VAD‐FMK group)

Next, z‐VAD‐FMK, the specific inhibitor of caspase, was employed to determine the potential necessary role of caspase‐dependent apoptosis during SEZ6L2 regulation of CRC cell growth. As shown in Figure 4A, the knockdown of SEZ6L2 significantly inhibited the cell viability of HCT116 cells, whereas z‐VAD‐FMK efficiently blocked the inhibition of shSEZ6L2 on HCT116 cell growth. Similar results were also found in the HT29 cells (Figure 4B). Furthermore, treatment with z‐VAD‐FMK also efficiently attenuated the inhibition of colony formation by shSEZ6L2 in HCT116 cells (Figure 4C,D). Collectively, caspase‐dependent apoptosis played a necessary role during SEZ6L2 regulation of CRC cell growth.

### 3.5 Knockdown of SEZ6L2 impairs CRC tumour growth in vivo

**Figure 5:**
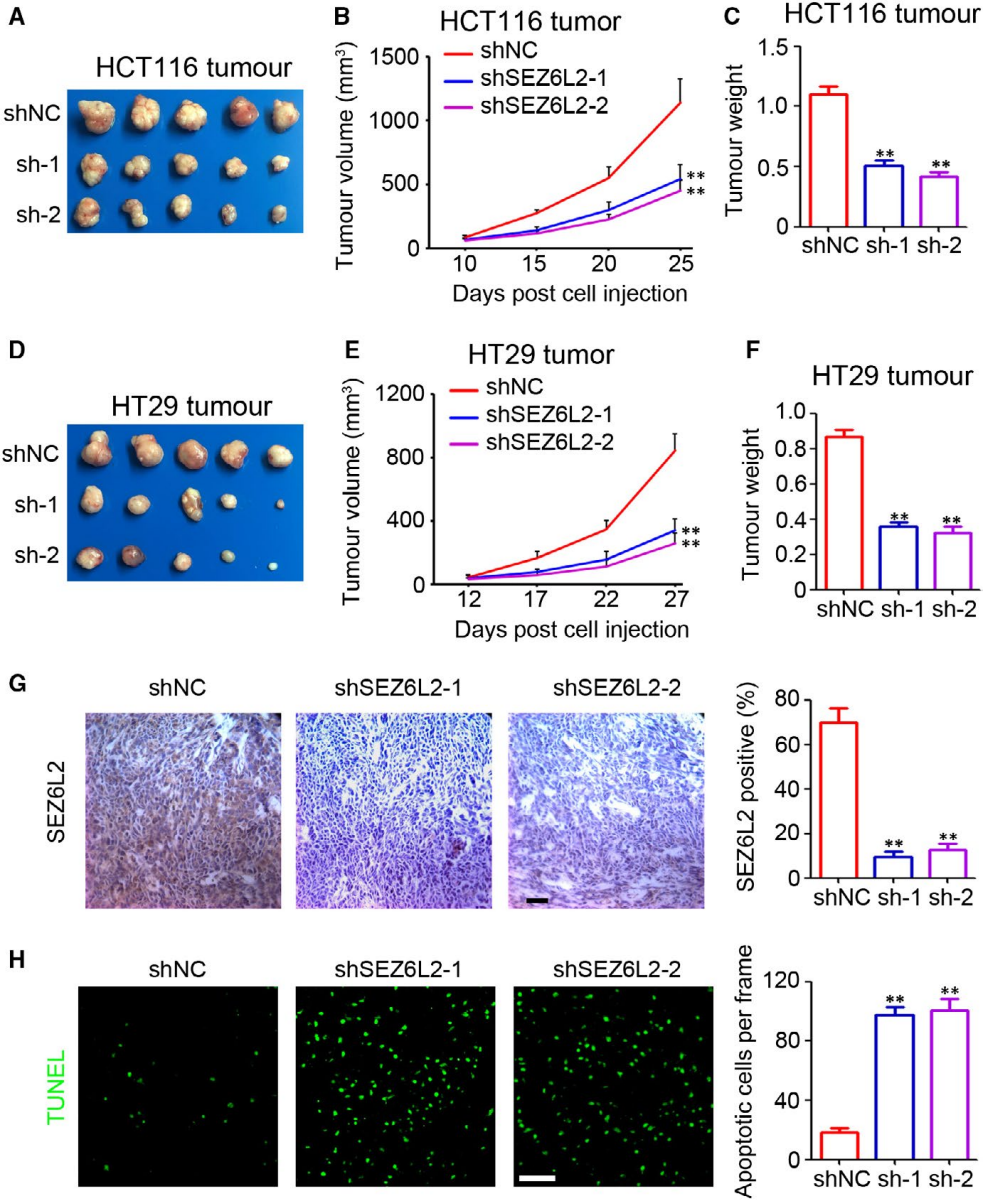
Knockdown of SEZ6L2 impairs CRC tumour growth in vivo. A‐C, Tumour image, tumour volume (n = 5, **P < .01) and end‐stage tumour weight (n = 5, **P < .01) after injection of 5 × 10^6^ HCT116‐shNC and HCT116‐shSEZ6L2 cancer cells into nude mice (25 d after cells injection). D‐F, Tumour image, tumour volume (n = 5, **P < .01) and end‐stage tumour weight (n = 5, **P < .01) after injection of 5 × 10^6^ HT29‐shNC and HT29‐shSEZ6L2 cancer cells into nude mice (27 d after cells injection). G, IHC staining of SEZ6L2 expression in HCT116 tumour tissues. Scale bar = 100 μm. The percentage of SEZ6L2 positive cells in tissues were analysed (n = 4, **P < .01). H, Analysis of apoptotic cells by TUNEL staining in HCT 116 tumour tissues. Scale bar = 100 μm. The number of apoptotic cells in each frame was counted and analyszed (n = 4, **P < .01)

To further determine the function of SEZ6L2 in CRC, HCT116 and HT29 cells were collected for the establishment of its xenograft model. Our results indicated that the knockdown of SEZ6L2 efficiently inhibited the volume of HCT116 tumours by 52.3% (shSEZ6L2‐1) and 60.1% (shSEZ6L2‐2) (Figure 5A,B, tumour volume: shNC group, 1138.6 ± 187.3 mm^3^ vs shSEZ6L2‐1 group, 543.2 ± 113.8 mm^3^ vs shSEZ6L2‐2 group, 453.8 ± 79.3 mm^3^). Similar inhibition of tumour weights was also found in the shSEZ6L2‐1 group (53.9%) and the shSEZ6L2‐2 group (62.1%) (Figure 5C). The knockdown of SEZ6L2 also dramatically inhibited the tumour volume end‐stage weight of HT29 tumours (Figure 5D‐F). The IHC staining results demonstrated the low expression of SEZ6L2 in HCT116 tumour tissues from the shSEZ6L2‐1 and shSEZ6L2‐2 groups (Figure 5G). The TUNEL assay indicated that the knockdown of SEZ6L2 significantly promoted the apoptosis of HCT116 tumour cells (Figure 5H). The above results suggested that the knockdown of SEZ6L2 impairs CRC tumour growth in vivo.

### 3.6 SEZ6L2 inversely correlates with cytochrome C expression in malignant tissues of patients with CRC

**Figure 6:**
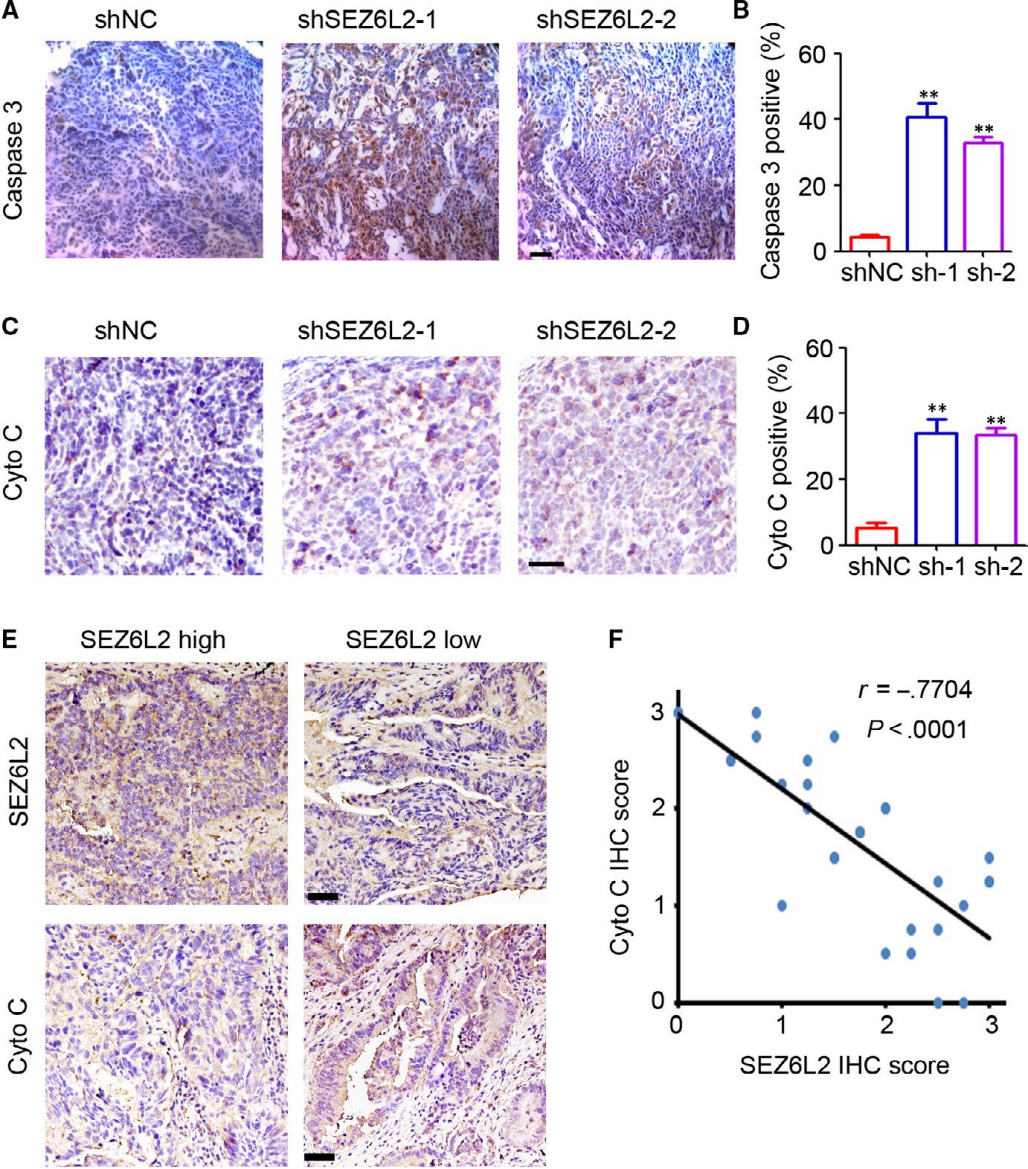
SEZ6L2 inversely correlates with cytochrome C expression in malignant tissues of CRC patients. A, IHC staining of caspase 3 expression in HCT116 tumour tissues. Scale bar = 100 μm. B, The percentage of caspase 3 positive cells in tissues were analysed (n = 4, **P < .01). C, IHC staining of cytochrome C expression in HCT116 tumour tissues. Scale bar = 100 μm. D, The percentage of SEZ6L2 positive cells in tissues were analysed (n = 4, **P < .01). E, IHC staining of SEZ6L2 and cytochrome C expression in malignant tissues of CRC patients. Scale bar = 100 μm. F, Pearson's correlation analysis of SEZ6L2 expression and cytochrome C expression (*r* = −.7704, *P* < .0001)

The expression of cytochrome C in tumour tissues of the HCT116 xenograft was determined by IHC staining. The results indicated that the knockdown of SEZ6L2 significantly promoted the expression of caspase 3 (Figure 6A,B) and cytochrome C (Figure 6C,D) in HCT116 tumours. Additional IHC staining of CRC tissues indicated a low expression of cytochrome C in SEZ6L2 high‐expression tissues and a high expression of cytochrome C in SEZ6L2 low‐expression tissues (Figure 6E). Pearson's correlation analysis confirmed the inverse correlation between SEZ6L2 expression and cytochrome C in CRC tissues (Figure 6F, *r* = −.7704). Collectively, SEZ6L2 is inversely correlated with cytochrome C expression in malignant tissues of patients with CRC.

## 4 DISCUSSION

Colorectal cancer and many other cancers are often the result of molecular alterations that lead to the enhanced function of oncogenes or loss of function of tumour‐suppressor genes.^15^ Suppression of the activator protein 1 (AP‐1), regulated by IFN (SARI), was demonstrated to be down‐regulated in CRC, and SARI expression was inversely correlated with poor clinical outcomes in patients with CRC.^16^ Furthermore, deregulated long non‐coding RNAs were also involved in regulating cell stemness, colon inflammation, oxidative stress response and cell death during CRC initiation and progression.^17^ SEZ6L2 expression in mouse embryos was restricted to the spinal cord and brain, while in the human foetal brain, it was highest in the post‐mitotic cortical layers, hippocampus, amygdala, and thalamus.^18^ SEZ6L2 expression was also up‐regulated in NSCLC.^12^ A higher level of SEZ6L2 expression was also correlated with shorter tumour‐specific survival of the patients.^12^ In the present study, we first demonstrated that SEZ6L2 expression was significantly up‐regulated in the malignant tissues of patients with CRC compared with paired adjacent normal tissues. Furthermore, the data from the TCGA also confirmed the up‐regulation of SEZ6L2 mRNA in CRC tissues. Analysis of the prognosis indicated that among patients with CRC, a high SEZ6L2 expression was correlated with a shorter overall survival time, which was demonstrated by our IHC and TCGA data. The inverse correlation between SEZ6L2 expression and the prognosis of cancer patients was also demonstrated in lung cancer.^12^ Thus, the present study suggested that SEZ6L2 is a potential prognosis biomarker for CRC patients.

Until now, the function of SEZ6L2 was poorly understood; however, studies have begun to reveal some of its basic functions. A previous study by Boonen et al^9^ suggested that SEZ6L2 is involved in the trafficking of cathepsin D to endosomes, and hence modulates neurite outgrowth. SiRNA‐mediated down‐regulation of SEZ6L2 prevents phosphorylation of adducin and neuritogenesis.^19^ Higher SEZ6L2 expression predicts a poor prognosis for NSCLC, but the function of SEZ6L2 in cancer was still unclear.^12^ In this study, the in vitro and in vivo results suggested that SEZ6L2 functioned as an oncogene in CRC. The knockdown of SEZ6L2 impairs tumour growth by promoting apoptosis, which indicates that SEZ6L2 could be a potential therapy target for CRC. However, our results demonstrated that SEZ6L2 has no significant effect on the invasion ability of CRC cells, which suggests that the metastasis of CRC would not be influenced by SEZ6L2.

Apoptosis was induced through caspase‐dependent and caspase‐independent pathways in diverse cells.^20^ We found that the knockdown of SEZ6L2 promotes the apoptosis of CRC cells by inducing the expression of caspase 3 and caspase 9 but has no significant impact on the expression of caspase 8. These results suggest that SEZ6L2 is not involved in the apoptosis mediated by Fas Ligand. Meanwhile, the balance among the Bcl‐2 family members, including pro‐apoptotic and anti‐apoptotic factors, determines the cell fate.^21^ Mitochondrial cytochrome c, which is regulated by Bcl‐2, induces apoptosis by promoting the expression of caspase 9 and caspase 3.^22^, ^23^ Bcl‐2 and Bax were important components of inducing intrinsic apoptosis, which responds to a series of apoptotic factors.^24^, ^25^ Our results suggested that the knockdown of SEZ6L2 promotes the expression of the pro‐apoptosis proteins Bim, Bax, and cytochrome C, while inhibiting the expression of the anti‐apoptosis protein Bcl‐2 in CRC cells. Notably, SEZ6L2 has no significant effect on the expression of BID in CRC cells. It is indicated that SEZ6L2 would be involved in the Bim‐mediated apoptosis which was regulated by the death stimuli signalling pathway.^26^, ^27^ We also confirmed the inverse correlation between SEZ6L2 and cytochrome C expression in the malignant tissues of patients with CRC. However, in the future, further investigations are needed to demonstrate the direct binding targets of SEZ6L2 when regulating caspase‐dependent apoptosis in CRC.

Collectively, our results demonstrated the prediction role of SEZ6L2 for the prognosis of patients with CRC and the antitumour effects of shSEZ6L2 in vitro and in vivo. The present study would provide a potential prognosis biomarker and therapy target for CRC. But further investigation is needed to demonstrated the direct binding targets of SEZ6L2, which may provide solid evidence for understanding the function of SEZ6L2 in cancers.

## CONFLICT OF INTEREST

All authors declare that there are no conflicts of interest.

## AUTHORS’ CONTRIBUTIONS

Ning An conducted all the experiments. Yaqin Zhao helped to draft the manuscript. Haitao Lan and Ming Zhang participated in the statistical analysis. Yuan Yin and Yi participated in the design of the study and finalized the manuscript. All authors read and approved the final manuscript.

## ETHICS APPROVAL AND CONSENT TO PARTICIPATE

All experimental procedures were approved by the Institutional Animal Care and Use Committees of Sichuan University.

## CONSENT FOR PUBLICATION

Consent for publication is not applicable in this study, and no individual person's data were used.
